# Opposing Effects on Vascular Smooth Muscle Cell Proliferation and Macrophage-induced Inflammation Reveal a Protective Role for the Proresolving Lipid Mediator Receptor ChemR23 in Intimal Hyperplasia

**DOI:** 10.3389/fphar.2018.01327

**Published:** 2018-11-20

**Authors:** Gonzalo Artiach, Miguel Carracedo, Joan Clària, Andres Laguna-Fernandez, Magnus Bäck

**Affiliations:** ^1^Department of Medicine, Karolinska Institutet, Stockholm, Sweden; ^2^Department of Biochemistry and Molecular Genetics, Hospital Clínic-IDIBAPS and Department of Biomedical Sciences, University of Barcelona, Barcelona, Spain; ^3^Theme Heart and Vessels, Division of Valvular and Coronary Disease, Karolinska University Hospital, Stockholm, Sweden

**Keywords:** intimal hyperplasia, macrophage, omega-3, resolution of inflammation, smooth muscle cells

## Abstract

Intimal hyperplasia remains a significant clinical problem in for example coronary artery bypass graft failure. Since omega-3 fatty acids reduce intimal hyperplasia, we hypothesized that the G protein-coupled receptor ChemR23 for the omega-3-derived pro-resolving lipid mediator resolvin E1 drives those effects. ChemR23^+/+^ and ChemR23^-/-^ mice were generated with or without introduction of the *Caenorhabditis elegans* *fat-1* transgene, which leads to an endogenous omega-3 fatty acid synthesis and thus increasing the substrate for resolvin E1 formation. ChemR23 deletion significantly increased intimal hyperplasia 28 days after ligation of the left common carotid artery. Mice expressing the *fat-1* transgene showed reduced intimal hyperplasia independently of ChemR23 expression. ChemR23^-/-^ Vascular smooth muscle cells (VSMCs) exhibited a significantly lower proliferation compared with VSMCs derived from ChemR23^+/+^ mice. In contrast, ChemR23^-/-^ peritoneal macrophages had significantly higher mRNA levels of pro-inflammatory cytokines compared with ChemR23^+/+^ macrophages. Finally, conditioned media (CM) transfer from ChemR23^-/-^ macrophages to VSMCs significantly increased VSMC proliferation compared with CM from ChemR23^+/+^ macrophages. Taken together, these results point to a dual effect of ChemR23 in resolution pharmacology by directly stimulating VSMC proliferation and at the same time suppressing macrophage-induced VSMC proliferation. In conclusion, these differential effects of ChemR23 signaling in VSMC and macrophages open up a novel notion for intimal hyperplasia pathophysiology, where ChemR23-transduced effects on the vascular wall may vary, and even be opposing, depending on the degrees of resolution of inflammation.

## Introduction

The effectiveness of drug eluting stents for the prevention of restenosis after percutaneous coronary interventions (PCI) relies on the potent effects of cell cycle inhibitors on vascular smooth muscle cell (VSMC) proliferation (Bäck and Hansson, 2015). Intimal hyperplasia, however, remains a significant clinical problem in for example coronary artery bypass graft failure (Wadey et al., 2018). In addition to direct effects on VSMCs, intimal hyperplasia is also driven by inflammation, by means of neutrophil, and macrophage infiltration, as well as cytokine and matrix metalloproteinase (MMP) release (Wadey et al., 2018). In particular, a failure in the resolution of the acute inflammatory response to vascular injury prevents re-endotheliazation and promotes VSMC proliferation and migration (Wu et al., 2017).

Omega-3 fatty acids decrease inflammation (Serhan, 2014), and inhibit intimal hyperplasia in mice (Li et al., 2015). Other studies have shown similar effects after administration of the pro-resolving lipid mediators resolvins and maresins, which are enzymatically formed from omega-3 fatty acids (Ho et al., 2010; Akagi et al., 2015; Liu et al., 2018; Wu et al., 2018). The latter studies provided the initial evidence that stimulating the resolution of inflammation by means of lipid pro-resolving mediators would promote an adequate healing of vascular injury. However, the receptor(s) involved in this protective effects *in vivo* remain unknown. We recently established a protective role for the G protein-coupled receptor ChemR23 for the omega-3-derived pro-resolving lipid mediator resolvin E1 in atherosclerosis (Laguna-Fernandez et al., 2018), but its implications for intimal hyperplasia have remained hitherto unexplored. The aim of the present study was therefore to establish the role of ChemR23 in the downstream signaling of omega-3 fatty acids in intimal hyperplasia, in a pro-inflammatory vascular injury murine model (Zhang et al., 2008).

## Methods

### Carotid Ligation

**FIGURE 1:**
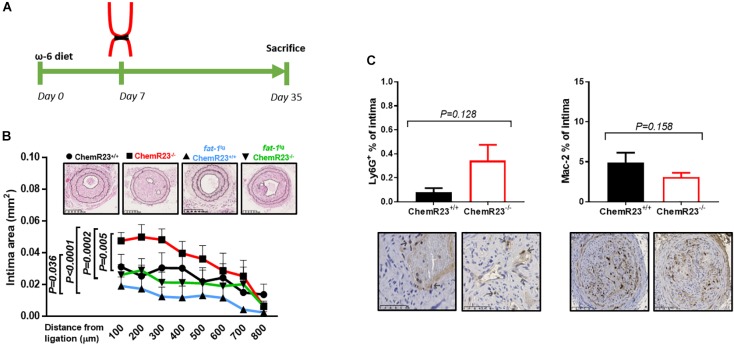
ChemR23 deletion promotes intimal hyperplasia under pro-inflammatory conditions. **(A)** Schematic representation of *in vivo* experimental procedure. **(B)** Mouse intima hyperplasia quantification in: ChemR23^+/+^ *n* = 7, ChemR23^-/-^ *n* = 7, *fat-1*^tg^ ChemR23^+/+^ *n* = 6, *fat-1*^tg^ ChemR23^-/-^ *n* = 8; after left common carotid ligation, and representative H&E stained photomicrographs. **(C)** Ly6G^+^ (neutrophil) ChemR23^+/+^ *n* = 5, ChemR23^-/-^ *n* = 7 and Mac-2 (macrophage) ChemR23^+/+^ *n* = 4, ChemR23^-/-^ *n* = 7 immunohistochemistry quantification, and representative photomicrographs. Data represent mean ± SEM. *P*-values derive from **(B)** 2-way ANOVA, **(C)** Student’s *t*-test.

The study was approved by the Regional Ethical Review Board in Stockholm. All animals used were male and on a C57BL/6J background. ChemR23^-/-^ mice were obtained from Deltagen. Mice expressing the *Caenorhabditis elegans* *fat-1*transgene were bred as previously described (Lopez-Vicario et al., 2015). The two mice strains were then crossbred to generate four groups; ChemR23^+/+^, ChemR23^-/-^, *fat-1*/ChemR23^+/+^, and *fat-1*/ChemR23^-/-^. 10 weeks old littermates (*n* = 6–8/group) were subjected to a complete left carotid ligation as previously described (Petri et al., 2015b) and indicated in Figure 1A. In brief, the four groups were fed in a 10% v/w Omega-6 (Sigma-aldrich, S8281) enriched diet to increase the *fat-1* desaturase substrate. EPA and DHA were quantified by gas chromatography as a control as previously described (Laguna-Fernandez et al., 2018), and exhibited the expected increase in *fat-1* transgenic mice independently of ChemR23 expression (data not shown). After 7 days, mice were anesthetized with isoflurane/O_2_ (2:1) followed by 0.1 mg *s.c.* injection of buprenorphine for pain relief. Left common carotid artery was exposed, followed by a complete ligation at the bifurcation level with a 7-0 suture. After 28 days from ligation, mice were euthanized by CO2, PFA fixated, and the ligated carotid was collected in PFA, and paraffin embedded. For carotid intimal hyperplasia evaluation, 8 sections of 10 um each every 100 um, were collected. Next, H&E staining was performed to assess neointimal growth area at the site of ligation. Furthermore, sections were stained with antibodies (Supplementary Table 1) for rabbit anti α-SMA (Abcam), rat anti CD206 (Serotec), rat anti Mac2 (Cedarlane), and rat anti Ly6G (BD) for macrophage and neutrophil content determination, respectively. Negative and isotype controls are shown in Supplementary Figure 2. Staining in the neointima was assessed using the automated software Leica QWin Standard Y 2.8 (Leica Microsystems) and normalized to neointima area. Rat and rabbit isotype controls were purchased from R&D, and Abcam, and secondary antibodies from Vector.

### VSMC Isolation and Evaluation of Proliferation

Abdominal aortas from ChemR23^+/+^ and ChemR23^-/-^ mice (*n* = 3/group) were isolated, fat and adventitia removed, and digested in a sterile mixture of 1 mg/mL collagenase type II (Worthington) and 0.3 mg/mL elastase (Sigma, E0127) in DMEM with 10% FBS for 90 min at 37°C and 5% CO_2_. Cell suspension was spun down, resuspended in complete medium (DMEM, 10% fetal calf serum, 100 units/ml penicillin, 100 μg/ml streptomycin, 1 mM sodium pyruvate, 10 mM HEPES, and 2 mM L-glutamine) and plated. Cells were passaged using trypsin when they reached 80% confluency. Proliferation was assessed by WST-1 reagent (Roche) according to manufacturer’s protocol.

### Peritoneal Macrophages Conditioned Media Generation

Peritoneal macrophages from ChemR23^+/+^ and ChemR23^-/-^ mice (*n* = 4/group) were obtained as previously described (Laguna-Fernandez et al., 2018). Macrophages were treated for 24 h with complete medium supplemented with LPS (100 ng/ml), washed and followed by a 24 h incubation in complete medium without LPS. After that, cell supernatant was collected and frozen. After thawing, supernatants were diluted 1:10 in complete medium and transferred to ChemR23^+/+^ VSMCs.

### RNA Extraction and Real-Time PCR

RNA from ChemR23^+/+^ and ChemR23^-/-^ peritoneal macrophages was isolated after 24 h of LPS (100 ng/ml) stimulation using the RNeasy Mini Kit (Qiagen). RNA concentration was quantified by Nanodrop (Thermo Scientific). Relative gene expression was assessed using Taqman assays from Life Technologies: GAPDH as endogenous control (Mm99999915), TNF-α (Mm00443258), IL-6 (Mm00443258), MMP9 (Mm00442991), IL-1β (Mm00434228), and IL-10 (Mm01288386).

### Statistics

Results are expressed as mean ± S.E.M. Statistical significance was assigned at *p* < 0.05 as assessed with Student *t-*test when comparing two groups, and with two-way ANOVA as appropriate followed by recommended *post hoc* tests, for multiple comparisons. All analyses were performed using GraphPad Prism 7 (GraphPad Software Inc., CA, United States).

## Results

We here report for the first time that genetic disruption of ChemR23 significantly increased intimal hyperplasia (Figure 1B). Furthermore, and as predicted, mice expressing the *Caenorhabditis elegans* *fat-1* transgene, which enables the endogenous production of omega-3 fatty acids, exhibited reduced intimal hyperplasia (Figure 1B). Crossbreeding of the two models also allowed us to determine the interaction between the observed effects attributed to omega-3 fatty acids and ChemR23, respectively. Unexpectedly, the *fat-1* transgene was protective in both ChemR23^+/+^ and ChemR23^-/-^ mice (Figure 1B). Immunohistochemistry in intimal lesions revealed an infiltration of neutrophil granulocytes (Ly6G) and macrophages (Mac-2), which were not significantly different between ChemR23^+/+^ and ChemR23^-/-^ mice (Figure 1C), whereas no T-lymphocyte infiltration (CD3) was detected (data not shown). Analysis of CD206, as a marker of M2 macrophages, revealed no significant differences between ChemR23^+/+^ and ChemR23^-/-^ mice in the percentage of CD206 positive cells in the intima (1,52 ± 0,47, 1.26 ± 0.38, *p* = 0.682), nor in the ratio between M2 and total macrophages (CD206/Mac-2) (31.17 ± 9.66, 40.98 ± 12.30, *p* = 0.538) (Supplementary Figure 1A). Finally, intimal lesions stained positive for α-smooth muscle actin, but revealed no differences in the percentage of α-smooth muscle actin in the intima between ChemR23^+/+^ and ChemR23^-/-^ mice (Supplementary Figure 1B).

**FIGURE 2:**
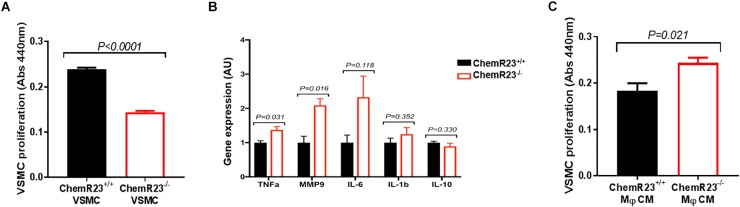
ChemR23 deletion alters the Vascular smooth muscle cells (VSMC) and macrophage phenotype. **(A)** Basal ChemR23^+/+^ and ChemR23^-/-^ VSMC proliferation (48h) *in vitro,* assessed by WST-1. **(B)** mRNA expression of LPS-activated (100 ng/mL, 24 h) peritoneal macrophages *in vitro*. **(C)** VSMC proliferation (48 h) treated with conditioned media (CM) derived from LPS-activated ChemR23^+/+^ and ChemR23^-/-^ macrophages. For VSMC *n* = 3/group, for macrophages *n* = 4/group. Data represent mean ± SEM. *P*-values derive from Student’s *t*-test.

To decipher the mechanisms involved in the phenotype associated with ChemR23-deficiency, we subsequently isolated abdominal aortic VSMCs from ChemR23^+/+^ and ChemR23^-/-^ mice. ChemR23^-/-^ VSMCs exhibited significantly less proliferation compared with ChemR23^+/+^ VSMCs (Figure 2A). These *in vitro* findings were hence in sharp contrast to the increased intimal hyperplasia observed in ChemR23-deficient mice *in vivo*. To further characterize the ChemR23-dependent phenotype, we subsequently studied the inflammatory response in LPS-activated peritoneal macrophages, isolated from ChemR23^+/+^ and ChemR23^-/-^ mice. ChemR23^-/-^ macrophages exhibited a more pro-inflammatory phenotype, with significantly higher mRNA levels of TNFα, and MMP9 (Figure 2B). These results indicated that in intimal hyperplasia, ChemR23^-/-^ VSMCs may have a protective phenotype (less proliferation), whereas ChemR23^-/-^ macrophages would be detrimental (increased inflammation). To assess the consequences of the differential macrophage phenotypes on VSMC proliferation, VSMCs were treated with CM derived from LPS-activated ChemR23^+/+^ and ChemR23^-/-^ macrophages, respectively. ChemR23^-/-^ macrophage CM significantly increased VSMC proliferation as compared with VSMC treated with ChemR23^+/+^ media (Figure 2C).

## Discussion

Three major observations emerge from the present study. First, our results revealed a protective role of ChemR23 in intimal hyperplasia, which was unrelated to the beneficial effects of enriching tissues with omega-3. Secondly, we identify ChemR23 as a transducer of opposing effects in different cell types, in terms of suppressing inflammatory activation of macrophages, but stimulating proliferation of VSMCs. Third, ChemR23^-/-^ macrophages in turn promoted VSMC proliferation. Taken together, these results suggest that ChemR23 signaling toward the resolution of inflammation protects from intimal hyperplasia by means of reducing macrophage activation.

The observation that genetic ChemR23 deletion increased the inflammatory response in macrophages supports previous reports (Cash et al., 2014; Lopez-Vicario et al., 2017) and is consistent with our recent findings that ChemR23 deletion in hyperlipidemic mice accelerated atherosclerosis (Laguna-Fernandez et al., 2018). The present study extends that observation by showing that a failure in the resolution of macrophage-induced inflammation by means of ChemR23 deficiency increased VSMC proliferation. This effect was independent of an increased total number of macrophages or M2 macrophage subtype. In accordance with this findings, in atherosclerotic lesions ChemR23 expressing macrophages do not correspond to an M2 phenotype (Laguna-Fernandez et al., 2018). Indeed, previous results indicate that ChemR23 expression in M1 macrophages acts to promote the resolution of inflammation (Herova et al., 2015). Altogether the expression of ChemR23 in both macrophages and smooth muscle cells in human atherosclerotic lesions (Laguna-Fernandez et al., 2018), support an extrapolation of these results to human pathophysiology.

Previous reports have shown that pro-resolving lipid mediators decrease VSMC proliferation (Wu et al., 2018), and that VSMC lacking the lipoxin, and D-resolvin receptor ALX/FPR2 exhibit decreased migration (Petri et al., 2015b). In the present study, however, ChemR23 deficient VSMCs proliferated less compared with wild-type VSMCs. Hence, in the absence of a macrophage-derived response, ChemR23 signaling may in contrast promote VSMC proliferation. These observations suggest that while ChemR23 signals to limit macrophage-induced inflammation, ChemR23 may transduce deleterious effects on VSMCs under non-inflammatory conditions. Whether different agonists transduce those differential responses, however, remains to be established. In general, pro-resolving agonists are associated with a beneficial smooth muscle response in vascular injury (Ho et al., 2010; Wu et al., 2018), abdominal aortic aneurysms (Pope et al., 2016; Petri et al., 2018) and atherosclerosis (Viiri et al., 2013; Petri et al., 2015a).

The reduced intimal hyperplasia by ChemR23 appeared independently of the beneficial effects of omega-3 fatty acids, and suggest ChemR23 expression being directly coupled to the VSMC, and macrophage phenotypes. Other studies have indeed implicated signaling through the free fatty acid receptor-4 to mediate beneficial effects of omega-3 fatty acids in and murine models of intimal hyperplasia (Li et al., 2015).

In conclusion, the opposing effects of ChemR23 on VSMC and macrophages reported in the present study raise a novel notion for intimal hyperplasia pathophysiology, namely that the same receptor may transduce both protective and deleterious effects, which may vary over time depending on different stages in the resolution of inflammation.

## Ethics Statement

This study was carried out in accordance with the recommendations and guidelines of the Regional Ethical Review Board in Stockholm. The protocol was approved by the “Regional Ethical Review Board in Stockholm.”

## Author Contributions

GA, MC, and AL-F designed and performed the experiments, and analyzed the data. MB, GA, MC, and AL-F conceived the study. GA, MC, and MB wrote the manuscript. All authors participated in the interpretation of the data and provided critical review of the manuscript.

## Conflict of Interest Statement

The authors declare that the research was conducted in the absence of any commercial or financial relationships that could be construed as a potential conflict of interest.
