# Supplementary material for: Opposing Effects on Vascular Smooth Muscle Cell Proliferation and Macrophage-induced Inflammation Reveal a Protective Role for the Proresolving Lipid Mediator Receptor ChemR23 in Intimal Hyperplasia
Source: Front Pharmacol. 2018 Nov 20;9:1327. doi: 10.3389/fphar.2018.01327 (PMC6255922; doi:10.3389/fphar.2018.01327)
Supplement: Supplementary file 1 [file Table_1.pdf]

**Supplementary material:**

**Supplementary Figure 1** Immunohistochemistry of α-smooth muscle actin (A) and CD206 (B) and representative photomicrographs.

**A)**

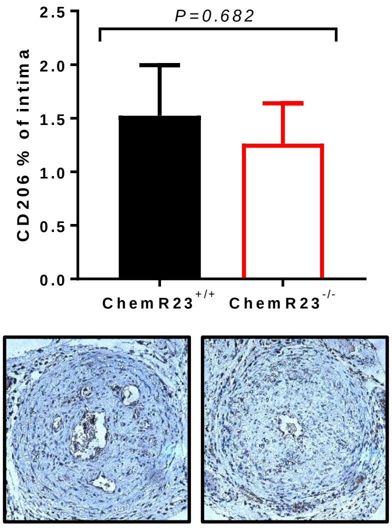


ChemR23$^{+/+}$ n=7, ChemR23$^{-/-}$ n=6

**B)**

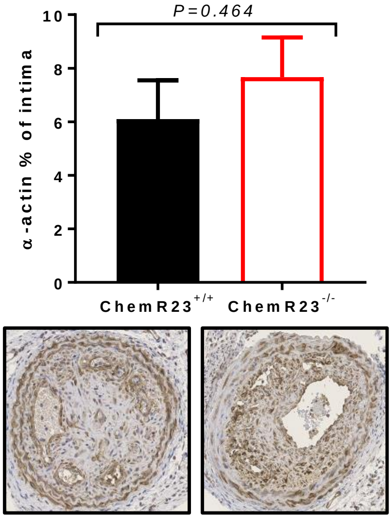


ChemR23$^{+/+}$ n=7, ChemR23$^{-/-}$ n=7

**Supplementary Figure 2** Immunohistochemistry controls

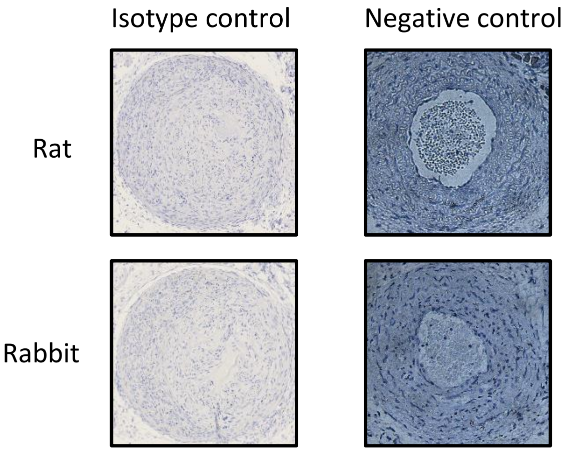

**Table 1: Antibodies and dilutions**

| Antibody | Species | Concentration | Vendor | Reference |
|---|---|---|---|---|
| α-SMA | Rabbit | 1:5000 | Abcam | Ab5694<br>Lot:GR24833630 |
| CD206 | Rat | 1:50 | Serotec | MCA2235<br>Clone: MR5D3 |
| Mac-2 | Rat | 1:2000 | Cedarlane | CL8942<br>Clone: M3/38 |
| Ly6G | Rat | 1:1500 | BD | 551459<br>Clone: 1A8 |
| Rat Isotype | Rat | 1:1500 | R&D | MAB006<br>Clone:54447 |
| Rabbit Isotype | Rabbit | 1:5000 | Abcam | Ab27478 |
| Anti-rat $2^{ary}$ | Goat | 1:200 | Vector | BA9401 |
| Anti-rabbit $2^{ary}$ | Goat | 1:200 | Vector | BA1000 |
